# Renal CIC-LEUTX rearranged sarcoma with multiple pulmonary metastases: a case report and literature review

**DOI:** 10.1186/s12882-023-03404-x

**Published:** 2023-11-30

**Authors:** Ying Tang, Xialiang Lu, Rui Zhan

**Affiliations:** Department of Pathology, Suzhou Ninth People’s Hospital, Soochow University, Ludang Road 2666#, Wujiang District, Suzhou, 215200 Jiangsu China

**Keywords:** CIC-rearranged sarcomas, Kidney, CIC-LEUTX

## Abstract

**Background:**

CIC-rearranged sarcomas (CRS) are a group of heterogeneous tumors which mostly occur in the soft tissues of limbs and trunk, and are highly invasive with poor prognosis. Here, we describe a rare case of CRS that occurred in the left kidney with a CIC-LEUTX rearrangement.

**Case presentation:**

A 45-year-old male was admitted to hospital with a dry cough for more than two months without obvious cause. Physical examination and laboratory tests revealed no notable abnormality. The CT scan demonstrated a mass in the left kidney and multiple nodules in both lungs. The percutaneous core needle biopsy showed similar histomorphology and immunophenotype of small round cell malignant tumors. Genetic test revealed a CIC-LEUTX gene fusion.

**Conclusions:**

We present a rare primary renal CRS with multiple pulmonary metastases, and LEUTX is confirmed as the fusion partner of CIC gene for the first time in a renal case.

## Background

CRS, categorized into the undifferentiated small round cell sarcomas in the fifth edition of the World Health Organization Classification of Tumors of Soft Tissue and Bone in 2020, are a group of malignancies with a high metastatic rate and poor chemo responses. Most tumors occur in the deep soft tissues of the extremities and trunk, and rarely in viscera, including brain, and bone [1]. Genetically, among CRS, the most frequent CIC rearrangement partner is DUX4 (comprising 95% of the cases), followed by FOXO4, LEUTX, NUTM1, and NUTM2A [2]. So far, approximately five cases with CIC-LEUTX gene fusion were reported and four of them are in CNS [3–6]. Here, we report for the first time a case of renal CIC-LEUTX rearranged sarcoma with multiple lung metastases.

## Case presentation

**Fig. 1:**
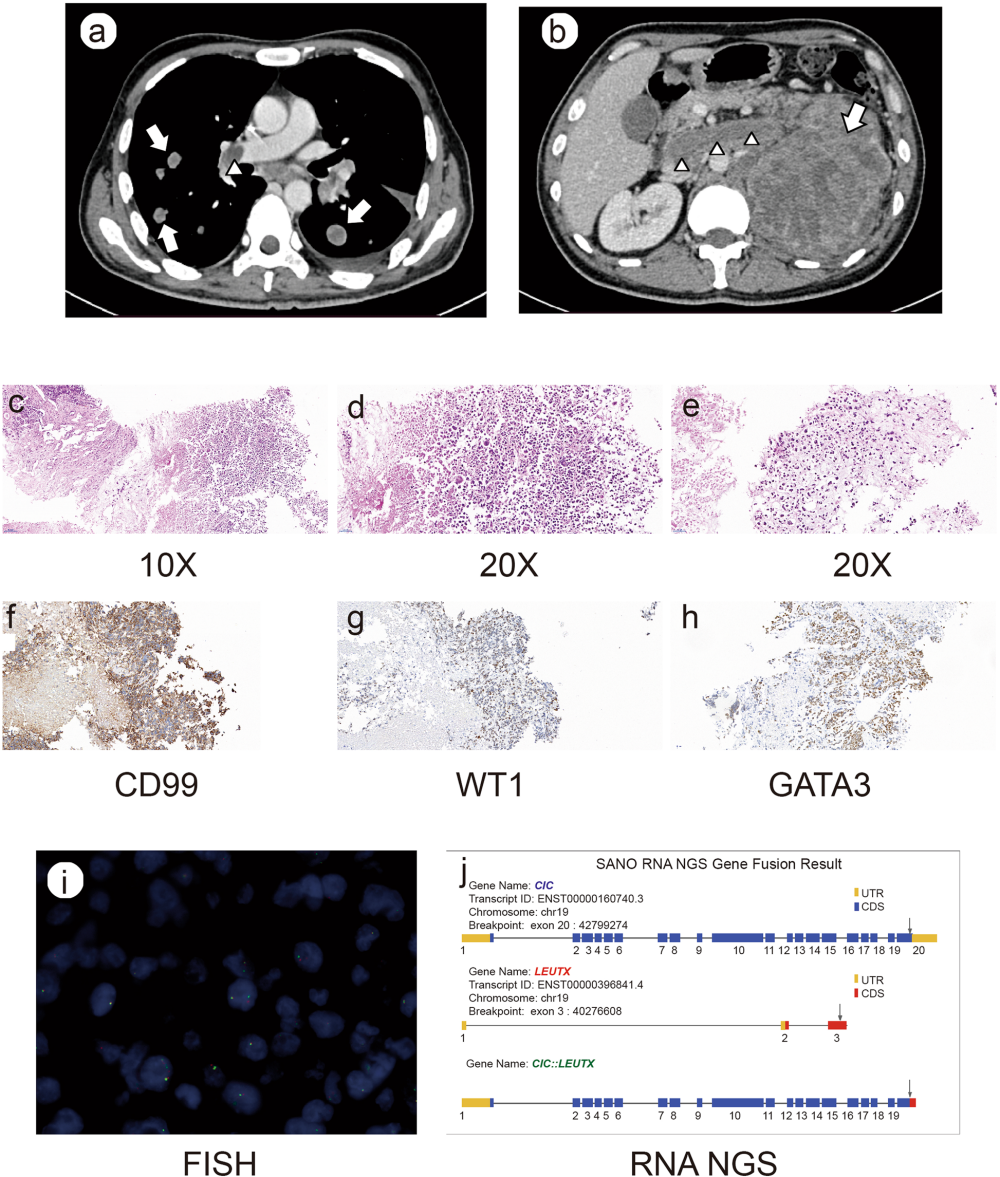
CT scan and pathological information of the case. **a** The chest CT scan showed multiple nodules (  ) in both lungs with filling defects (  ) in pulmonary arteries. **b** The abdominal CT scan showed an enlarged heterogeneous left renal mass (  ) replacing the left kidney and associated tumor thrombus (  ). **c-e** HE staining showed the tumor cells were organized in a solid sheets pattern (**c** 10x; **d** 20x). Large aeras of necrosis and partial myxoid stromal changes (**e**) were observed. **f–h** Immunohistochemical staining showed the tumor cells were positive for CD99 (**f**), WT1 (**g**) and GATA3 (**h**). **i** The fluorescence in situ hybridization assay showed separated red and green signals in the nuclei of 30% of tumor cells. **j** The next generation sequencing assay displayed a fusion of CIC-LEUTX genes. The breakpoints were at chr19: 42799274 for CIC and chr19: 40276608 for LEUTX, respectively

A 45-year-old male presented with cough for more than two months without obvious cause in August 2022. He claimed no other notable symptoms, and no evident abnormality was found in the laboratory blood test. Thus, infectious diseases were preliminarily excluded. For further examination, a chest CT scan (Fig. 1a) showed that there were multiple nodules in both lungs with enlarged hilar lymph nodes, and multiple filling defects in both pulmonary arteries, consistent with pulmonary thromboembolism It was considered as tumor metastasis. An abdominal CT (Fig. 1b) was performed for further evaluation. It showed an enlarged heterogeneous left renal mass almost completely replacing the left kidney (size 12.2 cm*9.7 cm), with mild perirenal fluid and fat stranding. There was associated tumor thrombus present in the left renal vein, along with enlarged metastatic retroperitoneal lymph nodes. The patient had a history of hypertension and diabetes, which were under well control with regular medication, and had no history of other diseases or surgery.

The patient underwent lung and kidney percutaneous core needle biopsy and the histological manifestations and immunochemistry (IHC) findings of the two samples are similar. Microscopically, the tumor cells were small to medium sized round or oval cells with poor adhesion. The cytoplasm was eosinophilic but scant, and the nucleoli were prominent with rough chromatin. The mitotic rate was high (> 10 mitotic figures per 10 high power field). Most of the tumor cells were organized in a solid sheets pattern. Large aeras of necrosis and partial myxoid stromal changes were observed (Fig. 1c-e). And in some aeras, the tumor cells were distributed around the open thin-walled vessels. Immunohistochemically, the tumor cells were focal positive for CD99 (Fig. 1f); multifocal positive for WT1 (Fig. 1g) with unequal strength, BRG1, focal weak positive for AE1/AE3 and diffuse positive for GATA3 (Fig. 1h), whereas S-100, Synaptophysin, Vimentin, CK7, PAX8 and TTF-1 were negative. The Ki67 proliferation index was high (> 80%). The fluorescence in situ hybridization (FISH) assay showed separated red and green signals or single red signals seen in the nuclei of 30% of tumor cells, suggesting the existence of CIC gene translocation (Fig. 1i). Moreover, the next generation sequencing (NGS) assay confirmed the existence of a fusion of CIC-LEUTX genes. The breakpoints were at chr19: 42799274 and chr19: 40276608 for CIC and LEUTX, respectively (Fig. 1j).

The patient received chemotherapy (paclitaxel + carboplatin) and immunotherapy(pabolizumab). However, brain metastases were found 2 months after diagnosis. Due to the rapid progression, a new chemotherapy regimen (Doxorubicin + vincristine + cyclophosphamide) and radiotherapy was applied. Unfortunately, treatment was not effective enough and the patient passed away 7 months after diagnosis.

## Discussion and conclusions

The most common primary renal malignant tumor is renal cell carcinoma of epithelial origin, while the mesenchymal and neuroendocrine originated tumors are extremely rare. In this case, the histologic morphology and IHC stainings that AE1/AE3 weakly positive, and CK7, PAX8 and Syn negative, suggested that the diagnosis of epithelial and neuroendocrine tumors was not valid. Because of the tumor cells were mostly small round cells and expressed CD99, WT1 and BRG1, and negative for Vimentin, the diagnosis of sarcomatoid carcinoma was excluded. The dilemma lies in Ewing or Ewing-like sarcomas. The absence of specific EWSR1-ETS fusion for the former, and existence of a CIC-LEUTX gene fusion leaded to the diagnosis of CRS.

CRS is the most common type of Ewing like sarcomas, represents a new entity that has morphological similarity but differs in IHC, genetic and clinical manifestation while comparing to Ewing sarcoma (EWS) [7]. Currently, there are about 200 cases reported around the world [8–21]. Generally speaking, it mostly occurs in young adults and children, but the olders can also be affected, with a slight male predominance. According to our study, 84% of the cases occurred in the deep soft tissue of the limbs and trunk, and most of them were in the deep muscle. About 14% occurred in the viscera, including the kidney, gastrointestinal tract, lung and brain. Very few cases occurred in the bone, mostly in the pelvis. Among them, the kidney and digestive tract are the primary sites with relatively high incidence, about 2/3 of the visceral CRS occurs in these two sites, and the lung and brain are the most frequent sites of metastasis. Interestingly, from the 8 cases of renal CRS (Table 1) that we can get detailed information, we found that the majority of tumors originated in the right kidney. Previously, only Mangray et al. [15] reported a primary renal case of left side in an 82-year-old woman.

**Table 1:** Reported cases of renal CIC-rearranged sarcomas

Literature /Year	Age/Sex	Side/Size	IHC CD99/WT1/ETV4	FISH /Sequence	Status (month)
Mangray et al. /2016 [16]	9/Male	Right/10 cm	+ / + /U	CIC + ; EWSR1-;SYT- / CIC-DUX4	DOD (18)
Bergerat et al. /2017 [9]	29/Male	Right/15 cm	+ / + /U	CIC U; EWSR1- / CIC-DUX4^a^	DOD (5)
Camille et al. /2018 [10]	12/Male	Right/7.8 cm	+ / + /U	CIC-DUX4 + ; EWSR1- / CIC-DUX4	DOD (17)
Mangray et al. /2018 [15]	13/Female	Right/12.5 cm	+ / ±	CIC-NUTM1 + / CIC-NUTM1	NED (36)
Mangray et al. /2018 [15]	33/Female	Right/18 cm	+ / ±	CIC + ; EWSR1- / U	DOC (6)
Mangray et al. /2018 [15]	82/Female	Left/5 cm	+ / + / +	CIC + ; EWSR1- / CIC-DUX4	DOD (6)
Mangray et al. /2018 [15]	48/Female	Right/4.6 cm	+ / ±	CIC U; EWSR1-;SYT- / CIC-DUX4	AWD (8)
This case	45/Male	Left/12.2 cm	+ / ±	CIC + / CIC-LEUTX	DOC (7)

*Abbreviations*: *AWD* alive with disease, *DOC* died of complications, *DOD* died of disease, *NED* no evidence of disease, *U* unknown

^a^RT-PCR results

Morphologically, CRS exhibit a more heterogeneous appearance when compared with EWS. The tumor cells usually organized in solid sheets or a lobulated growth pattern in sclerotic stroma, with scant cytoplasm and prominent nucleoli, and may contain focal spindle cells, epithelioid cells and myxoid stromal changes. Geographic areas of necrosis and hemorrhage are commonly seen, and mitotic count is usually high. In our case, the growth pattern and cell morphology of tumor are basically consistent with those reported in previous literatures. The expression of CD99, a hallmark of EWS, can be observed in approximately 85% of the CRS cases. However, it is often patchy with a cytoplasmic diffuse expression pattern, lacking the strong, diffuse membranous staining observed in EWS [22]. The expression of WT1 and ETV4 is consistently present, which is useful for the differential diagnosis of CRS, although not entirely specific. According to Hung et al. [12], the sensitivity and specificity of diffuse ETV4 expression for CRS are 90% and 95%, respectively, whereas the sensitivity and specificity of WT1 are 95% and 81%, respectively. CRS can also express Keratin, S100, Fli1, ERG, Calretinin, etc. in varying degrees, but they are not specific. The expression of other markers in our case is basically the same as that in the literature, while GATA3 is diffuse nuclear strong positive, which has not been reported before. GATA3 can be a useful tool in various tumors in kidney [23], however, whether it has differential diagnostic value for CRS needs to be confirmed by more cases. In the 8 reported cases of renal CRS, CD99 and WT1 were all positive, while ETV4 was only positive in 1 case, so it is not recommended to be a good auxiliary diagnostic indicator.

The identification of molecular features is now the standard to confirm the diagnosis of CRS. In clinic, FISH dual-color break-apart probes can be used to detect the rearrangement of CIC gene, but the negative result cannot completely rule out CRS. Yoshida et al. [20] demonstrated that the detection of FISH probe has a false negative rate of 14%, thus RT-PCR, tri-color FISH detection or second-generation sequencing can be added if necessary. By far the most frequent fusion partner is DUX4 (95% of cases), followed by FOXO4, LEUTX, NUTM1, and NUTM2A. The type of the chimeras may be relevant to their biological characteristics since, for example, CIC-NUTM1 sarcomas show distinct anatomic tropism for the axial skeleton and unfavorable behavior compared with classic CRS [24].

Among the other 7 cases of renal CRS reported so far, 5 were CIC-DUX4, 1 was CIC-NUTM1, and the other 1 case was unsequenced due to lack of materials. However, with its break-apart probe test positive, DUX4 staining positive, it tended to be a CIC-DUX4 fusion. Our case indicated a rare fusion of CIC-LEUTX by NGS sequencing. This fusion located in exon 20 of CIC gene and exon 3 of LEUTX gene, and the breakpoints were at chr19: 42799274 and chr19: 40276608, respectively. LEUTX is a member of the paired homeobox genes, which plays an important role in human embryonic development and is silenced postnatally. CIC-LEUTX cases seemed to have a preference for the central nervous system (CNS), and its onset age is very young. Of the 5 reported cases (Table 2) of CIC-LEUTX rearrangement, 4 occurred in the CNS and were younger than 19 years old, and only 1 case of 26-year-old female occurred in the soft tissue of the lower limb. In our case, no CNS lesion was found when searching for the primary tumor, combined with the manifestation of solitary renal mass, positive GATA3 staining, it was considered primary renal malignancy. But soon it developed brain metastasis 2 months after diagnosis.

**Table 2:** Reported cases of tumors with CIC-LEUTX fusion

Literature /Year	Age/Sex	Location/Size	Diagnosis	IHC	Molecular Change	Status(Month)
Huang et al./2016 [6]	26/Female	Thigh/7.5 cm	Angiosarcomas	Positive: CD31, ERG; Negative: CD99, S100, desmin	CIC-LEUTX	DOD(33)
Lake et al. /2020 [4]	19/ Female	Brain/U	Anaplastic ganglioglioma	Positive: synaptophysin, NSE	CIC-LEUTX	AWD(56)
Lake et al. /2020 [4]	12/ Female	Brain/U	Anaplastic astrocytoma with epithelioid GBM features	Positive: GFAP, CD34, synaptophysin,	CIC-LEUTX	AWD(3)
Hu et al. /2020 [3]	2/Male	Brain/7.5 cm	CNS embryonal tumor	Positive: synaptophysin Negative: GFAP	CIC-LEUTX	AWD(11)
Song et al. /2022 [5]	16/Male	Spinal Cord /1.2 cm	CIC-rearranged sarcoma	Positive: CD99, WT1, nestin, synaptophysin, D2-40	CIC-LEUTX	AWD(5)
This case	45/Male	Left kidney/12.2 cm	CIC-rearranged sarcoma	Positive: CD99, WT1, BRG1, GATA3, AE1/AE3; Negative: S-100, Vimtenin, synaptophysin, PAX8, TTF-1	CIC-LEUTX	DOC(7)

*Abbreviations*: *AWD* alive with disease, *DOD* died of disease, *DOC* died of complications, *U* unknown

Currently, patients with CRS are routinely treated in the same way as EWS, with a neoadjuvant and adjuvant anthracycline-based polychemotherapy regimen, surgery, and radiotherapy [1]. The CRS generally has a dismal prognosis. The 5-year overall survival is around 50%, which is significantly lower than the 80% 5-year overall survival of EWS patients [8]. This may also be related to the metastatic rate of about 50% at the time of initial diagnosis [8]. Limited Data indicate that CRS are less chemo-sensitive than EWS [1], and the challenging aspect remains to design more specific novel therapies. Yoshimoto et al. [25] found that palbociclib and trabectedin can block the growth of CIC-DUX4 sarcomas in an ex vivo mouse model. Carrabotta et al. [26] provides proof of principle for combination therapy with trabectedin and AKT/mTOR dual inhibitors to combat CIC-DUX4 sarcomas in the experimental models of patient-derived xenografts (PDX) and PDX-derived cell lines. These studies provide us with encouraging possibilities for the treatment of CRS, but optimal treatment remains to be further clarified, and more clinical data and experimental results are required.

To the best of our knowledge, this is the first case of CIC-LEUTX rearranged sarcoma that occurred in the kidney. Although the primary site is extremely rare, its histomorphology and immunophenotype are similar to those of other common sites. The unreported positive staining of GATA3 may be one of the diagnostic indicators for the kidney origin. The final diagnosis depends on FISH detection or sequencing of CIC gene rearrangement. This tumor showed a poor outcome and overall survival was dismal with no effective treatment. More clinical data and laboratory results are need to clarify the biological characteristics, and develop more targeted treatment accordingly.

## Data Availability

All data related to this case report are within the manuscript.

## Abbreviations

CRS: CIC-rearranged sarcomas
IHC: Immunochemistry
FISH: Fluorescence in situ hybridization
NGS: Next generation sequencing
EWS: Ewing sarcoma
CNS: Central nervous system
PDX: Patient-derived xenografts
